# Ethnobotanical and economic value of *Ravenala madagascariensis* Sonn. in Eastern Madagascar

**DOI:** 10.1186/1746-4269-10-57

**Published:** 2014-07-15

**Authors:** Nivo Rakotoarivelo, Aina Razanatsima, Fortunat Rakotoarivony, Lucien Rasoaviety, Aro Vonjy Ramarosandratana, Vololoniaina Jeannoda, Alyse R Kuhlman, Armand Randrianasolo, Rainer W Bussmann

**Affiliations:** 1 Missouri Botanical Garden, Madagascar Research and Conservation Program, BP 3391, Antananarivo 101, Madagascar; 2 Department of Plant Biology and Ecology, University of Antananarivo, BP 906, Antananarivo 101, Madagascar; 3 William L. Brown Center, Missouri Botanical Garden, P.O. Box 299, St. Louis, MO 63166-0299, USA

**Keywords:** *Ravenala madagascariensis*, Ambalabe, Madagascar, Ethnobotany, Uses, Conservation

## Abstract

**Background:**

Known worldwide as the “traveler’s tree”, the Malagasy endemic species *Ravenala madagascariensis* Sonn. (Strelitziaceae) is considered as an iconic symbol of Madagascar. It is a widespread species in the eastern part of the country with four different varieties which are well represented in Ambalabe community. All of them are used for different purposes and the species represents an important cultural value in the lives of the local population. However, uses of Ravenala are only generally well known by local population. Thus, in this study, we report on the different uses of *Ravenala* and its importance to the Ambalabe local people.

**Methods:**

Semi-structured interviews among 116 people, 59 men and 57 women with ages ranging from 17 to 84 years old, free listing and market surveys were conducted in order to collect the vernacular names, the uses of *Ravenala madagascariensis* and the price of plant parts sold in local market. Then, the uses were categorized according to Cámara-Leret *et al.* classification.

**Results:**

Different parts of the plant are currently used by local population, which are grouped as heart, trunk, leaves, petioles and rachis. Seven categories of use were recorded, most cited include: human food, utensils and tools, and house building. The most commonly used parts are trunk, heart, leaves and petioles for which the price varies between $3-15. Uses mentioned for construction (floor, roofs and wall), human food and utensils and tools are the most frequent and salient for local population. But the use of the plant as first materials for house building is revealed to be the most important for them.

**Conclusions:**

*Ravenala madagascariensis* is very important to the Ambalabe communities because for local population, it represents the Betsimisaraka cultural and traditional use of the plant for house building. Moreover, none of its parts are discarded. The harvest and sale of *R. madagascariensis* for building materials can also provide an additional source of income to the family. Besides, using *Ravenala* in house construction reduces the use of slow growing trees and contributes to the sustainable use of natural forest resources.

## Background

**Figure 1:**
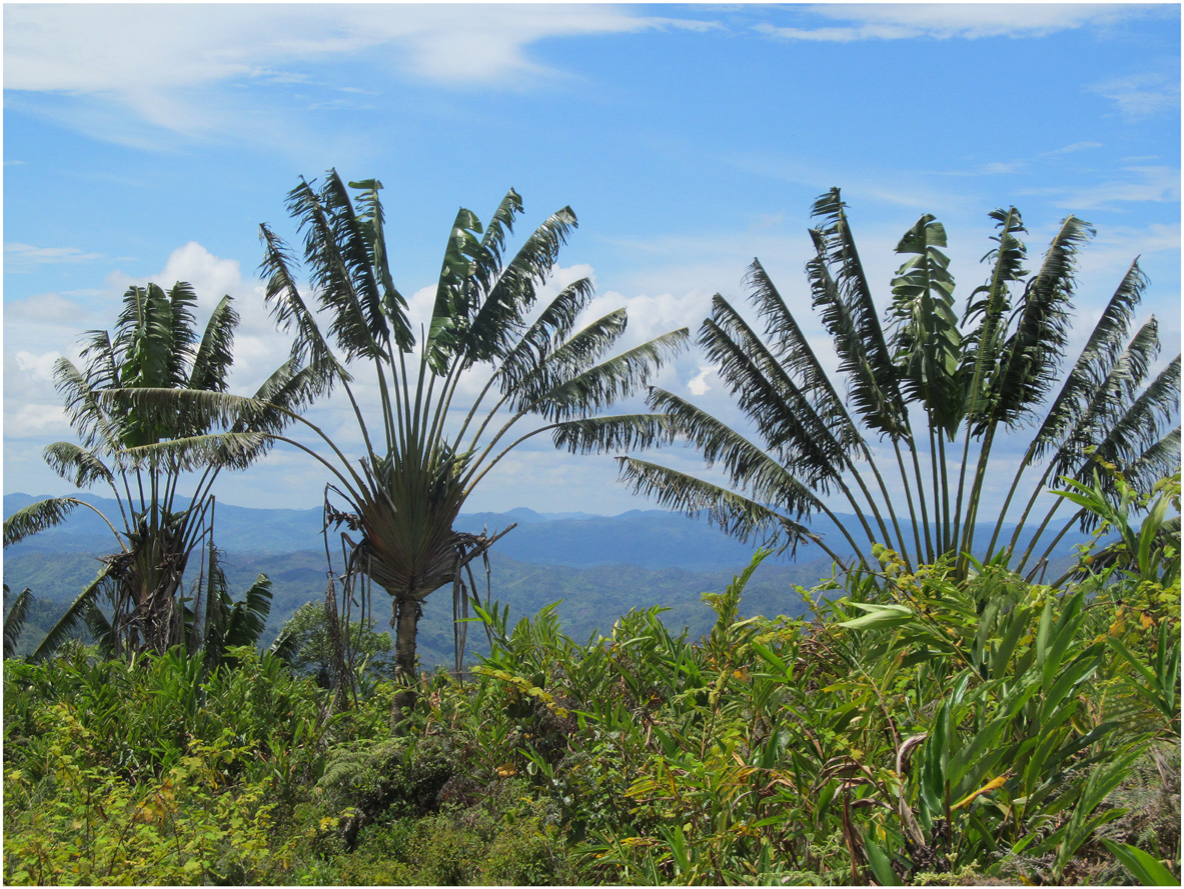
**Pictures of *Ravenala *** **tree**

**Figure 2:**
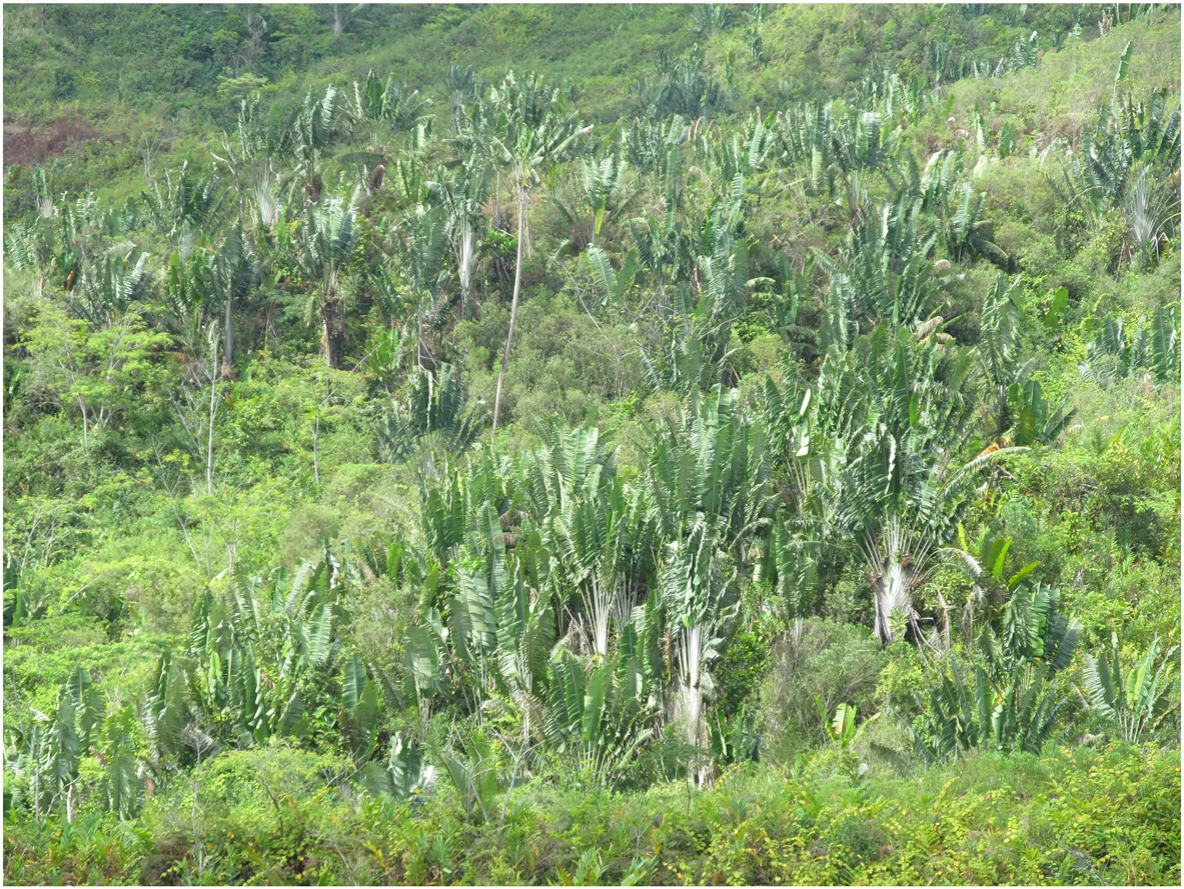
**Population of *Ravenala madagascariensis *** **called « *Ravenala *** **forest ».**

Madagascar has a remarkable wealth in terms of vegetation and endemic species. The island is composed of a multitude of natural environments [1] which harbor a unique and globally important assemblage of plant species [2]. In the eastern part of Madagascar, *Ravenala madagascariensis* (Figure 1), an endemic species also known as the traveler’s palm or traveler’s tree, is considered as an iconic symbol of the island. While *Ravenala madagascariensis* occurs in both primary rainforest and open secondary growth [3], it is known to form a very characteristic vegetation called “*Ravenala* forest” [4], due to the high abundance of the species (Figure 2).

**Figure 3:**
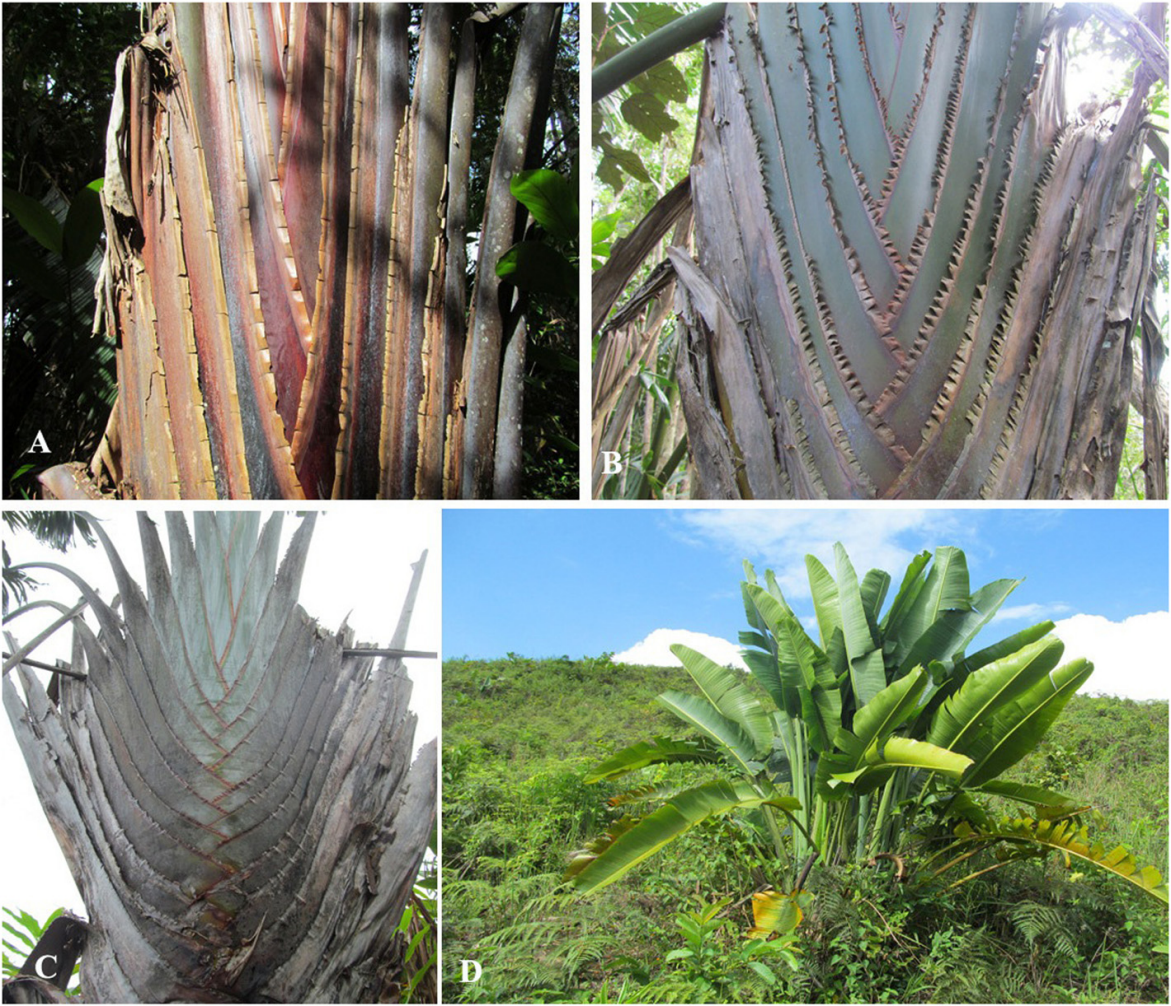
**Pictures of the four varieties of *Ravenala *found in Ambalabe. (A)***Menafalaka*: found only in the forest, the picture shows its red sheath. **(B)***Hirana*: occurs within and outside the forest, picture showing the sheath borded by fringes. **(C)***Bemavo*: grows on deforested slopes, with the petioles covered with gray powders. **(D)***Horonorona*: picture showing its cespitose habit.

Always regarded as monospecific, *Ravenala madagascariensis* has recently proved to be a complex aggregate of at least four recognized varieties, which might deserve the rank of species [5]. Each variety can be distinguished according to macromorphological characters, growth habits and habitat preferences [6,7]. Four varieties of *Ravenala* are well represented in Ambalabe community in Eastern Madagascar. Three of them match the three recognized vernacular names cited by Blanc *et al*. in 2003, such as *Hirana* (known before as *menahirana*, from which *mena* means *red* and *hirana* means *fringes* because it can be easily recognized by the sheath which is borded by red fringes), *Bemavo* (*be* means *many* and *mavo* means *gray* in Betsimisaraka language because of the gray powder along the sheath) and *Horonorona* (the name comes from its cespitose habit which is in tuft). The variety *Hirana* occurs both inside and outside Vohibe forest which belongs to Ambalabe commune, and the two varieties *Bemavo* and *Horonorona* are only encountered outside the forest. The fourth one which only occurs inside Vohibe forest has a red sheath and is locally called *Menafalaka* (*mena* means *red* and *falaka* means *sheath* in Malagasy) (Figure 3).

*Ravenala madagascariensis* is well known as an ornamental plant and is cultivated in many tropical regions. Aside from ornamental value, *Ravenala* appears to have many uses [4,8,9]. In the Ambalabe community, the four varieties are all used by local population. Our purpose was to report on the different uses of *Ravenala* and to assess its ethnobotanical and economic value for the local population.

## Methods

This study is conducted within a framework of an existing collaboration between the local population and the staff at the Missouri Botanical Garden Ambalabe Conservation Project. Fieldwork was conducted from October-December 2013 in order to collect vernacular names, the plant use and the local price of the used plant parts. A community meeting was held to explain the objective of the study in order to obtain a prior consent from the administrative authorities, traditional leaders and the local population. The surveys were done in the presence of a local guide and were conducted in the local dialect of Malagasy.

### Study Site

**Figure 4:**
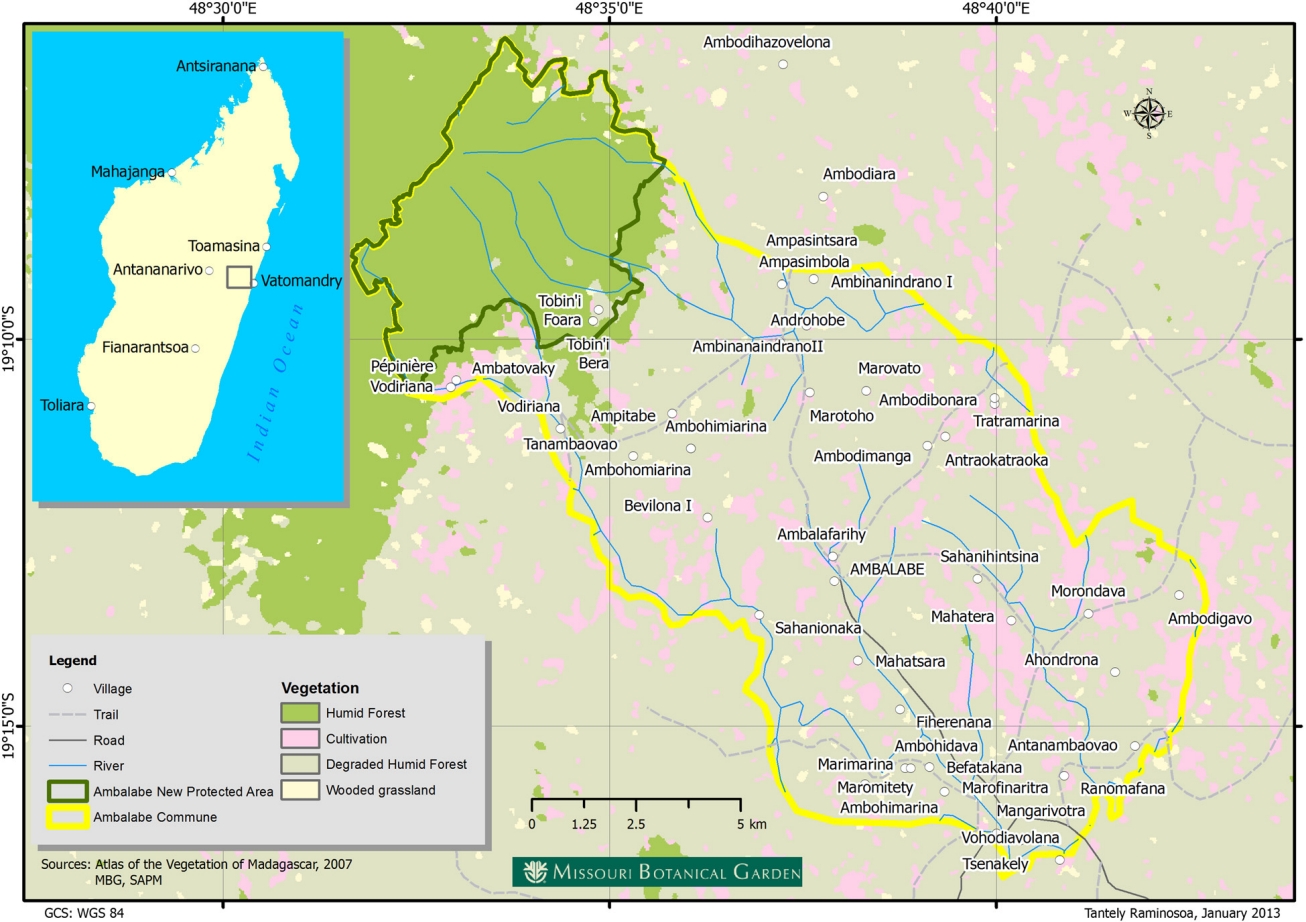
Location of Ambalabe rural commune.

This study was conducted in the rural commune of Ambalabe, 72 km northwest of the district of Vatomandry, which is one of the 7 districts of the Atsinanana Region in Eastern Madagascar and the nearest large city and marketplace (Figure 4). Ambalabe is a very remote area, and is only accessible by road via the Provincial Interest Road (RIP) N°8, linking it to Vatomandry via Antanambao Mahatsara. The road is only passable in the dry season from August to Obtober by 4x4 vehicles up to Ambodinonoka (46 km from Vatomandry). Local people, for cost and accessibility reasons, prefer walking or using wooden canoes via Sakanila River from the village of Befatakana to Tsarasambo, or sometimes use motorcycles from the village of Vohidiavolana to Vatomandry. The whole commune covers 17,437 ha including the forest area, with a population of 10,961 inhabitants in 2013, of which 95% are farmers. About 42 villages and 1,437 households are recorded within the commune (rural commune of Ambalabe, personal communications).

The rural commune of Ambalabe is characterized by rough topography, with valley systems with steep slopes of 10-60%, and narrow, almost reduced bottomlands. This topographical system of alternating mountain and valley typically represents the landscape in this part of Madagascar. The community is subject to a humid tropical climate [10] with an average annual rainfall of 1773 mm, distributed over 214 days. The average annual temperature is 24°C (75°F).

### Ethnobotanical surveys

Due to deficiencies in roads and to the rough topography, local populations rely on the use of plants in their daily life. *Ravenala madagascariensis* is one of the most important plant resources used by local communities in Ambalabe. In order to know the different uses of *Ravenala* and to assess its importance, semi-structured interviews and market surveys were carried out within villages after obtaining prior informed consent [11-14] from local and tribal leaders and survey participants. Questionnaires about *Ravenala* were established and used as a guide for the surveys and informants were asked to cite uses they know (free list) for *Ravenala* and the price of materials they use (Additional file 1). Interviews were conducted in Malagasy local dialect by the four first authors, who are native speakers. A local guide acted as a dialect translator as necessary. Field walks with some participants were also done in order to identify the four varieties of *Ravenala* and pictures were taken. Then, uses were categorized according to Cámara-Leret *et al.*[15] use category.

**Table 1:** **Different uses of *Ravenala madagascariensis *** **recorded among the local people in Ambalabe with their frequency**

**Use category**	**Uses**	**Parts used**	**Frequency (%)**	**Salience**
Human food	Cooked as vegetable	Heart	57.8	0.394
Animal food	Fodder for domestic animals (zebu)	Heart	1.7	0.003
Construction	Floor	Trunk	64.7	0.461
	Roofs	Leaves	50	0.404
	Wall	Petioles and trunk	42.2	0.303
	Beehives	Trunk	3.4	0.024
	Chicken coops	Leaves	1.7	0.012
	Gable	Trunk	0.9	0.009
	Doors	Petioles	0.9	0.003
Environmental uses	Cultivated in the garden as ornamental plant	Whole plant	0.9	0.002
Medicinal and veterinary	Dizziness	Young leaves	1.7	0.011
	Stomach-ache	Young leaves	1.7	0.006
	Regulates albumin levels	Heart	0.9	0.003
Utensils and tools	Ropes and moorings	Petiole fibers	25.9	0.112
	Winnowing trays	Petioles	15.5	0.066
	Spoons	Leaves	9.5	0.016
	Baskets	Petioles	3.4	0.03
	Fishing nets	Leaf rachis	3.4	0.027
	Mats	Petioles	2.6	0.017
	Cupboard	Trunk	0.9	0.006
	Chicken baskets	Petioles	0.9	0.005
	Beds	Trunk	0.9	0.004
	Mat for food and market display	Fresh, whole leaf	0.9	0.004
	Dishes	Leaves	0.9	0.004
	Tables	Trunk	0.9	0.002
Other uses	Hosts edible larvae	Trunk	0.9	0.006

### Analysis

Data analysis was performed using the ANTHROPAC® 4.0 software package [16,17] and XLSTAT®-Pro 7.5. In this study, ANTHROPAC® was used for the analysis of free listing data. The results were expressed as frequency (%) and salience (a value that lies between 0 and 1). Frequency is the repetition of mentions during the surveys, of which one specific use of one plant part by one informant is counted as one mention. Salience is a statistic accounting for rank and frequency of the uses (e.g., one use is more salient when it appears more often and earlier in freelists) [18]. Thus, uses that are frequently mentioned are assumed to be highly salient to respondents, and uses recalled first are assumed to be more salient than uses recalled last [19]. Most frequent and most salient use of *Ravenala* is then considered as the important use of the species for the local population.

XLSTAT®-Pro 7.5 was used for the analysis of variance or ANOVA in order to see the difference between men and women knowledge on the uses of *Ravenala*.

## Results and discussion

### Ethnobotanical Knowledge

Thirteen villages were visited during the ethnobotanical surveys and 116 people were interviewed, of which 59 (51%) were men, and 57 (49%) women. The age of informants ranges from 17 to 84 years old. *Ravenala madagascariensis* is generally known locally as *Fontsy*, but there are four varieties encountered in Ambalabe known as *Menafalaka*, *Hirana*, *Bemavo* and *Horonorona*. Three of them, such as *Hirana*, *Bemavo* and *Horonorona* are already recognized by Blanc *et al.*[5].

In general, uses mentioned by participants during the free listing exercise correspond to the local name *Fontsy*. The species is used as human food, animal food, medicine, house building, tools and utensils, for environmental purposes, and also for other uses (Table 1). The frequency and the salience of each use cited were given with the table. However, some specific uses were attributed by participants to each variety depending on the used plant parts (Table 2). The plant is also known to provide seed arils and nectars which are eaten by lemurs and birds [3], and its leaves have an antidiabetic activity [20].

**Table 2:** **Specific uses of the four varieties of *Ravenala madagascariensis *** **according to surveys done in Ambalabe**

**Local name**	**Use category**	**Uses**	**Parts used**	**Abundance**
*Bemavo*	Human food	Cooked as vegetable	Heart	High
	Construction	Wall	Petioles and trunk	
		Floor	Trunk	
		Roofs	Leaves	
	Utensils and tools	Ropes and moorings	Petiole fibers	
*Hirana*	Human food	Cooked as vegetable	Heart	Rare
	Construction	Floor	Trunk	
	Medicinal and veterinary	Regulates albumin levels	Heart	
*Horonorona*	Construction	Floor	Trunk	High
		Roofs	Leaves	
*Menafalaka*	Construction	Floor	Trunk	Rare
		Roofs	Leaves	

**Figure 5:**
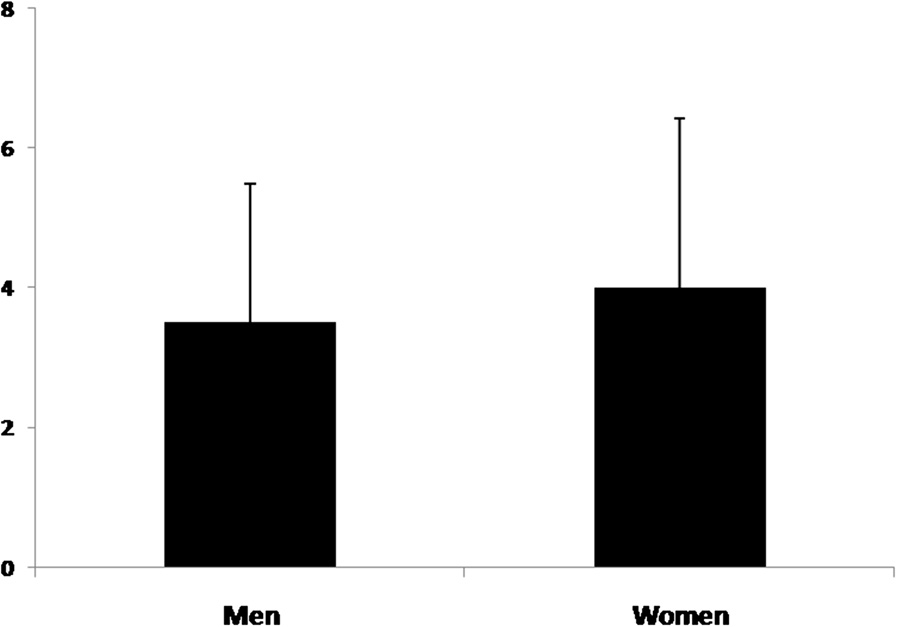
**Knowledge of men and women on *R. madagascariensis *** **uses.**

Twenty-six types of use of *Ravenala* were recorded. Women knew more uses (22 cited) than men (18 cited), however the analysis of variance shows that no significant difference were shown from the two genders (Figure 5), with P = 0.6 > 0.05. For both, the most frequent and salient uses cited are: floor, food, roofs, wall and rope.

All parts of the *Ravenala* tree are used by local people. The heart of *Ravenala*, locally called *Ovitra* of the two varieties *Bemavo* and *Hirana*, is edible and can be eaten by people or livestock. The variety *Bemavo* is widely preferred because it is sweeter. The variety *Hirana* is slightly bitter but people occasionally eat it [8]. *Ravenala* is used for food especially during “starvation period”, and the heart of the plant is often used as fodder during this period as well.

In terms of construction, flattened pieces of the trunk of all varieties are used for floor, but *Bemavo* is the most used because it has the biggest trunk. To make roofs, leaves of three varieties can be used, but *Horonorona* is widely preferred due to its longevity. However, using the species for rope is much more frequent because it is used whenever local people tie something, even for housing.

**Figure 6:**
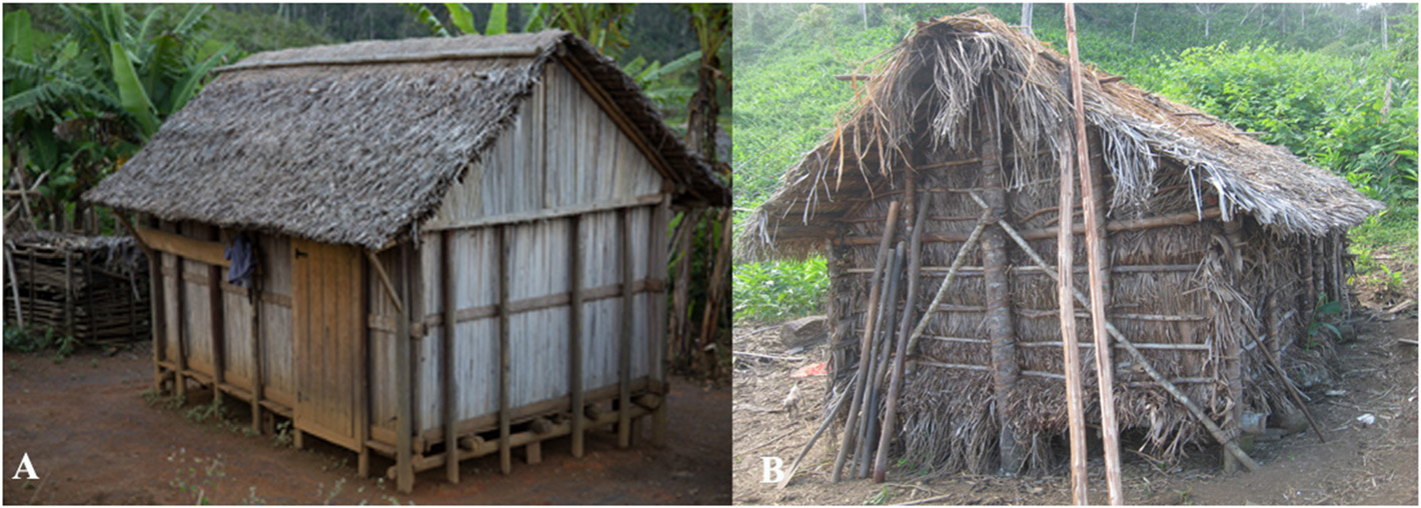
**Housing structures built with *R. madagascariensis. *(A)** Traditional house structure seen in Ambalabe using petioles for wall. **(B)** Temporary house structure built in the crop field with leaves used for roofs and wall.

The analysis of the use of *Ravenala* (Table 1) shows that the species is mostly mentioned for house building and food. As assumed by Collins *et al.*[21], we considered that uses which received the highest number of mentions are the most prevalent in the communities and also of the greatest importance to people living in the villages. This means that *Ravenala* is primarily used as first materials for construction in Ambalabe commune, in addition to other construction materials like wood for posts or rafters. Indeed, it is almost the common use of the plant in the East Coast of Madagascar and villagers depend on *Ravenala* for shelter [22]. In Ambalabe community, the species is used for traditional housing which is encountered in most villages, and also for temporary houses in the field in which leaves are also used for walls (Figure 6). The heart of the species is also used as substitute for food. It is a food rich in minerals [8]. Frequency for each use of *Ravenala* was considered in this study. The salience takes in account simultaneously the number and the order of citation.This analysis highlights the important use of *Ravenala* in house construction (floor, roofs, wall), and for food.

### Economic Value

As far as the economic value of *Ravenala* is concerned, we only considered the two varieties, *Bemavo* and *Horonorona*, which are encountered outside the forest. These varieties are more easily accessible for local population than the two others which mostly occur inside Vohibe forest. Besides, the use of plants from the forest is regulated by community rules called *dina*. For example, plants used for house construction, beehives, medicine, ferment for local alcohol drink, or dead trees for fuel can be taken from the part of the forest which is intended for traditional uses. However, those used for house construction, beehives and ferment always need authorization from the president of the local community-based regulation committee (locally called Vondron’Olona Ifotony or VOI) who regulates the use of forest products. If the trees are used for personal purpose, duty should be paid in advance in order to get the authorization, which can last for 3 months. If needed, it can be renewed after this period. Furthermore, it is noted that plants taken from the forest cannot be sold, but for personal use only. Thus, leaves, petioles and trunk of *Bemavo* and *Horonorona* are the parts sold in the market. Table 3 represents the variation of the price of the plant parts used to build houses and the expected longevity of the materials. An example of a house with 12 m^2^ floor area was taken.

**Table 3:** Estimated prices and longevity of plant materials used to build a house of 4x3 m

**Parts used**	**Local name**	**Part of house**	**Number needed**	**Total price**	**Longevity**
Leaves	*Bemavo*	Roofs	5-6 bundles of 100	$ 4-5	3 years
	*Horonorona*			$ 8-10	3-5 years
Petioles	Both	Wall	4-5 bundles of 100	$3-10	5-10 years
Trunks	*Bemavo*	Floor	8 pieces	$ 13	6 years
	*Horonorona*		12 pieces	$ 15	5 years
Fibers	Both	Mooring of roof or walls		~ $1	5 years

In this table, the price and the longevity depend on the type of materials needed and the variety of *Ravenala* used. *Horonorona* lasts for roofs while *Bemavo* for floor and wall. Materials need to be periodically renewed, usually at least every 3 years. Even though local populations are aware of the material’s short life span, they still use *Ravenala* because culturally it is the primary materials used for house building in the Betsimisaraka tribe. It is then a way to identify and preserve this culture. In addition, local population do not know how to manufacture bricks (personal communications), yet the plant is still abundant and easily accessible. Besides, it is an easy material to work with, allowing for fast house construction, which is particularly meaningful in Eastern Madagascar where cyclone damages are quite frequent [23]. In addition, *Ravenala* is a renewable material, and is inexpensive. Local populations also can directly take for them the materials they need without buying from others.

On the other hand, *Ravenala* provides a source of income which can improve or stabilize some people’s life. The raw materials (leaves, petioles, and trunk) can be sold directly to people in need who cannot go to collect the materials, and handicrafts made from *Ravenala*, such as winnowing trays or baskets, can be sold in local markets.

While *Ravenala madagascariensis* is very important for local population in Ambalabe community, not only in terms of uses (first material for house building) but also due to its affordable economic value, it is currently very difficult to find mature trees due to slash and burn cultivation, which is still widely practiced in this area [24,25]. Although the number of planted trees is abundant, it takes 15 to 20 years of growth to have a mature trunk for construction. Thus, people resort to the use of younger trees, from which the materials do not last for a long time, which means that the use of *Ravenala* might become more frequent, and the price might be increased. To tackle the issue, some management practices have been adopted by the local population. For example, they avoid logging *Ravenala* trees with trunks more than 1 meter in height when practicing the slash and burn cultivation, and field rotation was adopted for crops.

## Conclusions

*Ravenala madagascariensis* remains an important component in the life of local population in the Ambalabe Rural Commune, especially for house building. There are four varieties of *Ravenala madagascariensis* found in the study area and all of them are used. *Ravenala* is used primarily for construction, but other uses have also been noticed including food, medicine and tools. Using *Ravenala* for house building reduces the pressure on some forest trees, which contributes to the conservation of natural forests and slow growing hardwoods. However, mature trees are needed to source construction materials, and these have become increasingly scarce. While the local population has developed some practices to increase the numbers of large trees, strategies for long term management and sustainable harvests need to be developed.

## Competing interests

The authors declare that they have no competing interests.

## Authors’ contributions

All authors participated in the study design and drafted the manuscript. NR, AR, FR and LR carried out the study and analyzed the data. All authors read and approved the final manuscript.

## Supplementary Material

Additional file 1 Fiche d’enquête ethnobotanique. Click here for file
