# Supplementary material for: Ethnobotanical and economic value of Ravenala madagascariensis Sonn. in Eastern Madagascar
Source: J Ethnobiol Ethnomed. 2014 Jul 15;10:57. doi: 10.1186/1746-4269-10-57 (PMC4106185; doi:10.1186/1746-4269-10-57)
Supplement: Additional file 1 — Fiche d’enquête ethnobotanique. [file 1746-4269-10-57-S1.docx]

**Additional file 1**

**Fiche d’enquête ethnobotanique**

**N° informateur** :

Date : Village :

PROFIL PERSONNEL

Nom : Fonction :

Sexe : Masculin Féminin Age : Situation matrimoniale : Mariée Célibataire Veuf

Niveau d’étude :

INFORMATION SUR RAVENALA

1. Noms vernaculaires connus :
2. Signification des noms vernaculaires utilisés :
3. Caractères distinctifs de chaque variété :
4. Abondance de chaque variété :
5. Lieu de prélèvement :
6. Utilisations, parties utilisées, mode de récolte :
7. Mode de prélèvement de chaque partie utilisée :
8. Age de *Ravenala* exploitable (feuilles/pétioles, tronc, cœur) :
9. Utilisation de l’espèce : a) actuellement b) antérieurement
10. Quantité obtenue par pied et prix local de chaque partie :

- Feuilles obtenues par pied : Prix d’un paquet de 100 en ariary :
- Pétioles obtenus par pied : Prix d’un paquet de 100 en ariary :
- Plancher obtenus par pied : Prix de chaque pièce en ariary :
- Kilogramme d’un cœur pour un pied : Prix en ariary :

1. Quantité nécessaire de chaque partie utilisée pour construire une maison :
2. Longévité de chaque partie utilisée :
3. Raison de l’utilisation de *Ravenala* pour la construction :
4. Mode de gestion pour assurer l’utilisation à long terme de *Ravenala* :
